# The surfacing portion of the Iceberg of the Domestic Violence Phenomenon—data from the Israeli National Trauma Registry

**DOI:** 10.1186/s13584-021-00499-1

**Published:** 2021-12-02

**Authors:** Bella Savitsky, Irina Radomislensky, Sharon Goldman, Arielle Kaim, I. T. G. (Israel Trauma Group), A. Acker, N. Aviran, H. Bahouth, A. Bar, A. Becker, A. Braslavsky, D. Fadeev, A. L. Goldstein, I. Grevtsev, I. Jeroukhimov, A. Kedar, A. Korin, M. Qarawany, A. D. Schwarz, W. Shomar, D. Soffer, M. Stein, M. Venturero, M. Weiss, O. Yaslowitz, I. Zoarets, Moran Bodas

**Affiliations:** 1 Israel National Center for Trauma and Emergency Medicine Research, Gertner Institute for Epidemiology and Public Health Policy Research, Sheba Medical Center, 52621 Tel-Hashomer, Ramat Gan, Israel; 2 School of Health Sciences, Ashkelon Academic College, Ashkelon, Israel; 3 Department of Disaster Management, School of Public Health, Tel Aviv University, Tel Aviv, Israel

**Keywords:** Domestic violence, Violence against women, Injury, Trauma registry

## Abstract

**Background:**

Domestic violence against women, which is an ancient phenomenon, is still thriving worldwide. The burden of domestic violence that is non-fatal on scene and its consequences in Israel are unknown. The purpose of this study was to provide evidence-based data regarding domestic violence-related hospitalizations among women in Israel.

**Methods:**

The study is a retrospective cohort study of hospitalized patients included in the Israeli National Trauma Registry between January 1, 2011 and December 31, 2020. All women aged 14 and older, hospitalized due to a violence-related injury in one of the six-level I Trauma Centers or one of the 15 regional Trauma Centers in Israel were included (n = 676).

**Results:**

Domestic violence contributes to moderate, severe, and critical injuries in a quarter of abused hospitalized women. Among these women, 20% underwent surgery, and in-hospital mortality was recorded for 2% of the patients. For most cases (53%), the spouse or ex-spouse caused the injury. The family relationship with the perpetrator was distributed differently between the population groups. The proportion of brothers who attacked sisters was greatest among Arabs (14.4%), while the phenomenon of attacking a mother was infrequent in the Arab sub-group. In contrast, among Jewish women, the proportion of those injured by a son was high, especially among the group of Jewish immigrants from the Former Soviet Union (FSU) (17%) and other countries (26%). In a multivariable logistic regression model with at least moderate injury as a dependent variable, in comparison to Israeli Arabs, Jews had a higher odds for sustaining at least moderate injuries, while the odds of Jewish immigrants not from FSU or Ethiopia were the highest (OR = 4.5, 95% CI 2.0–9.9). The annual hospitalization risk was 1.3/100,000 and 5.8/100,000, respectively for Jews and Arabs in 2020, almost fivefold higher among Arab women in comparison to Jewish women (RR = 4.6, 95% CI 2.9–7.3).

**Conclusions:**

Domestic violence prevention should pay special attention to populations at risk, such as Arab women and new immigrants, as those women are especially vulnerable and often without sufficient family support and lack of economic resources to exit the trap of domestic violence. The collaboration between social and health services, the police, and the local authorities is crucial.

## Introduction

Domestic violence against women, an ancient phenomenon, is still thriving worldwide [1, 2]. An Ecological Model has been used to conceptualize violence as a multifaceted phenomenon grounded in an interplay among personal, situational, and sociocultural factors [3]. The model is based on four levels: primary, microsystem, exosystem, and macrosystem. The first level ("primary" or "individual") represents personal factors which a person brings to the relationship. The second level ("microsystem" or "family") represents the close relationships. The third level ("exosystem" or "community") represents formal and informal institutions (i.e., identity groups, neighborhoods, social networks). The last level ("macrosystem" or "society") represents cultural norms [4]. In this context, risk factors for violence against women may be easily classified. In relation to the primary level, being the victim of abuse or witnessing family violence is most strongly associated with becoming an abuser [5]. In the microsystem (family) level, risk factors for violence against women are related to male dominancy (for example, when the male head of the household controls all decisions regarding family finances), family conflicts, and heavy alcohol consumption by men [4]. The exosystem-related risk factors include unemployment or low socio-economic status (SES) and isolation of women, for example through limited support from the family and friends. Economic conditions are known triggers of violence. Underprivileged populations disproportionally account for the public health burden of violence in almost every society [6], and strong associations have been found between domestic violence and low household income, low educational level, consumption of alcohol, and drugs [5, 7, 8].

Finally, macrosystem factors include the broad set of cultural beliefs and attitudes which often support violence against women. The use of violence as a mean of control is percieved as more legitimate in patriarchal societies, where men are expected to hold control (especially over women), and violence allows to obtain and maintain that control [9]. The links between gender-based power and domestic violence are widely recognized, with violence being viewed both as a manifestation of deeply entrenched gender power inequities as well as a mechanism by which such inequities are enforced [7]. According to the U.S. Centers for Disease Control and Prevention (CDC), at least 5 million acts of domestic violence occur annually to women aged 18 years and older in U.S. [10]. About 35% of females experience some form of physical injury related to Intimate Partner Violence (IPV), and the data from U.S. crime reports suggest that over half of female homicide victims are murdered by a current or former male intimate partner [10]. A meta-analysis of 29 studies assessing the scope of domestic violence against women aged 15 years and above who accessed healthcare in Arab countries, produced pooled prevalence estimates of lifetime exposure to any type of IPV of 73%, physical IPV—36%, sexual IPV—22%, and emotional/psychological IPV—50% [11].

Israel is a country of contradictions and is comprised of a diverse population. On the one hand, the country is a Western-style democracy where a woman was prime minister, another was awarded the Nobel Prize in chemistry, another was the president of the Supreme Court [12], and females, like males, enlist in compulsory army service. On the other hand, a Jewish woman cannot be granted a divorce without her husband's permission [13], and according to Samah Salaime, a social worker who founded the Arab Women in the Center organization to aid victims of violence, "Israeli authorities treat the oppression of women as a value in Arab society" [14].

Jews from different cultural backgrounds and various degrees of religiosity comprise 75% of the population, and Israeli Arabs comprise almost 21% of the Israeli population [15]. The Arab culture is based on a patriarchal society where women are often expected to be dependent on their husbands, obey them, satisfy their needs, and take care of the children and maintain the household [16].

A study which compared Jewish and Arab women who were staying in shelters for victims of domestic violence concluded that Arab women, in comparison to their Jewish counterparts, were exposed to more physical abuse, received less family support, and the perpetrators were more likely to have access to weapons [17]. In a study conducted in 2015 among a sample of women visiting maternal and child health clinics, after adjustment for income and level of religiosity, Arab women had a 4.5 times higher rate of IPV compared to Jewish women [18].

In addition to the cultural diversity of Arabs and Jews, Jewish immigrants from around the globe have also contributed to cultural and behavioral diversity in Israel [18]. The most significant proportion of recent Jewish immigrants in Israel (comprising 20% of the total Jewish population) came from the Former Soviet Union and Africa (mainly Ethiopia) [19]. The findings of Sela-Shayovitz [20] suggest that between 1995 and 2007 in Israel, intimate femicide rates were much higher among immigrants in comparison to Israeli-born groups. Additionally, this study found that among immigrants from the Former Soviet Union (FSU), a significant proportion (almost one-third of cases) of the femicide cases were conducted under the influence of alcohol [20]. In this study [20], differences were found between the ethnic groups concerning the motive for femicide: among Israeli-born Jews the most frequent reason for femicide was the wife’s desire for separation (42.6%), whereas the dominant motive among Israeli Arabs was the wife’s alleged infidelity (60%). Among immigrants from FSU, 50% of femicide occurred during the course of an argument between the partners [20].

The Ethiopian community is another unique ethnic minority living in Israel, which includes approximately 85,000 Israeli citizens [21]. Not only has this ethnic community experienced challenges in social integration and absorption into Israeli society [22], but its origins are in a patriarchal society. In recent years, a high incidence of violence-related injuries requiring hospitalization have been reported among this population group [23, 24]. Economic distress experienced by Ethiopian immigrants has been associated with violence and abuse towards females [25]. This was supported by the findings in the femicide study, which reported that the motive for 45.8% of the femicide cases in this ethnic group was due to economic problems [20].

In 2020, the outbreak year of the Covid-19 pandemic and its related lockdowns, a second epidemic, one of violence against women, was witnessed. Since the beginning of the lockdowns in March 2020, a 75% increase in domestic violence was reported in Israel, according to Naila Awad, director-general of the Women Against Violence organization. The organization received about 700 testimonies of domestic violence during the first half of the year, compared to 800 reports throughout the entire year of 2019 [26]. The Women’s International Zionist Organization (WIZO) announced that its Center for the Prevention and Treatment of Domestic Violence recorded a 300% increase in the number of referrals and an increase of about 250% in the number of patients since the first lockdown earlier this year, compared to the same period the previous year [26]. Calls to WIZO’s emergency line for women affected by violence rose by about 75%.

Since the beginning of 2021, three outstanding cases of family violence were exposed through the media. In one case, Diana Raz, mother of four, was murdered by her husband, a police officer who first used a knife and then shot one bullet into her thigh and a second bullet to her head [27]. The second victim, Shira Isakov, mother of one, survived the extremely violent attack by her husband following her intent to leave him. Shira's husband stabbed her with a knife 20 times, leaving wounds on various parts of her body [28]. In the third case, Tatyana Kaminsky, a 53-year-old woman, an immigrant from Ukraine, was murdered by her 29-year-old son, and according to the neighbor, the mother suffered from recurrent acts of violence instigated by her son [29]. Due to the exposure of these and other violent cases in the media, there has been an increase in public awareness regarding violence against women.

Femicide represents only the tip of the iceberg of violence against women in Israel: while more than 20 women are killed annually by a family member (mostly spouses) [30, 31], only half of the victims had filed a violence-related complaint with the police prior to being murdered [31]. While the cases resulting in death receive national attention, the burden and consequences of the cases which result in physical and mental trauma receive little attention, and the phenomenon is less known.

The purpose of this study was to analyze domestic violence-related hospitalizations among women. The results will provide evidence-based data regarding domestic violence in Israel and increase awareness of the problem among healthcare professionals in an effort to identify abused women and reduce violence-related injuries and mortality.

## Methods

The study is a retrospective cohort study of hospitalized patients included in the Israeli National Trauma Registry (INTR) between January 1, 2011 and December 31, 2020. The INTR provides comprehensive data on hospitalized trauma patients from all six Level I trauma centers (TC) and 15 Level II TCs in Israel. All hospitalized trauma patients classified with an ICD-9-CM diagnosis code 800-989.9 who were admitted to the Department of Emergency Medicine (ED) and hospitalized, died in the ED or were transferred to or from another hospital are included in the database. The registry does not include casualties who died at the scene of the event or on the way to the hospital; and admissions 72 or more hours following the event. The data are recorded by trained trauma registrars at each TC under the supervision of a trauma director. Electronic files are transferred to the INTR, where quality assurance is carried out prior to data analysis. Data in the INTR is anonymous.

### Exclusion criteria

Military or terror-related injury, occupational trauma, suicide attempts.

### Inclusion criteria

All women aged 14 and older, hospitalized due to a violence-related injury in one of the six-level I TC's or one of the 15 regional TC's in Israel and recorded in the INTR were included.

The victims of family violence were identified by searching the free text of the injury description. The following variables were created manually:

*Domestic violence* yes (if the free text included an accurate description of the incident appropriate for domestic violence)/no (if the free text included an accurate description of robbery, brawl between neighbors or rape)/unknown (if the free text did not include an accurate description of the event or included a description of the brawl without specification who took part in the conflict).

*Perpatrator* male spouse or ex-spouse (husband, boyfriend, ex-husband, ex-boyfriend)/father/brother/ son/ other or unknown relative.

*Pregnancy* at the time of event: yes/no.

### Standard study variables

*Age* was used as a continuous variable.

*Population group* Immigrants from Former Soviet Union (IFSU)/Israeli born Jews (IBJ)/Immigrants from Ethiopia (EI)/Other Jewish Immigrants (OI)/Israeli Arabs (IA)/Foreign workers and tourists (FW)/Other or unknown.

*Injury Severity Score (ISS)*—the sum of the squares of the single highest Abbreviated Injury Scale (AIS) score for each of the three most severely injured body regions [32] categorized 1–8 (mild)/9–14 (moderate)/16 + (severe or critical injury) [33].

*Intensive Care Unit (ICU) stay*: yes/no.

*Trauma type:* Blunt/Penetrating/Burn.

*Injured body region with AIS* ≥ *3 (8 categories):* (1) Head and neck isolated injury; (2) Isolated face injury; (3) Isolated chest injury; (4) Isolated abdomen injury; (5) Isolated limb injury; (6) Isolated external injury; (7) Injuries with AIS severity 1–2 only (nobody region injured with *AIS* ≥ *3*); (8) *Multiple injuries* (two or more body region with *AIS* ≥ *3*).

*Length of Stay (LOS):* was used as a continuous variable.

### Statistical analysis

A univariate analysis examined the association between population group and injury characteristics using χ^2^ test, ANOVA test for the differences in the age, or Kruskal Wallis non-parametric test for differences in LOS.

The risk of being hospitalized following domestic violence was calculated using the data on the Israeli population in 2019 according to the Central Bureau of Statistics (2,730,200 Jewish women and 660,800 Arab women aged 14 years and older) [34].

Multivariable analysis with logistic regression approach was used with at least moderate injury (ISS 9+) as a dependent variable. Age, population group, perpetrator relation to the victim, and trauma type were included in multivariable analysis (correlation between the variables were checked with Kendall's Tau coefficient and all correlations were weak, with the strongest reaching Kendall's Tau coefficient of 0.09).

Analyses were carried out with SAS V.9.4 statistical software. For all analyses performed, a value of *p*<0.05 was considered statistically significant.

This study was approved by the ethical committee of the Sheba Medical Center.

## Results

During the study period, a total of 1637 women were hospitalized due to a violence-related injury; of them, 41.3% (n = 676) were victims of domestic violence, 28.0% (n = 458) were victims of a brawl between neighbors, robbery at home/on the street or rape. Among 30.7% (n = 503), the exact cause of the incident (domestic or other crime) was unknown.

This study focused on those women (n = 676) who were identified as victims of domestic violence. Among them, 654 were Israeli citizens, and 22 were foreign workers or tourists. Among 676 hospitalized casualties, 12.4% (n = 84) sustained moderate injuries (ISS 9–14), 11.1% (n = 75) suffered from severe or critical injuries (ISS 16 +) and 2% (n = 13) died during hospitalization.

Victims of domestic violence who immigrated to Israel from the FSU and OI were significantly older than victims from other population groups (mean age 50.9 and 66.3 years, respectively for FSU and OI, compared to 30.8–42.4 years for other population groups) (Table 1). Immigrants from the FSU, aged 72 years and older, comprised 17%, and among other Jewish immigrants comprised 44%. The mean age of Arab victims was the lowest (30.8 years), and the proportion of girls aged less than 18 years among Arabs was 17% (vs. 5.3% among Jews, *p* < 0.0001).

**Table 1:** Hospitalized victims of domestic violence, demographic and injury characteristics by population group, 2011–2020

Characteristics	IBJ	IFSU	EI	OI	IA	FW	Other or unknown	Total
n	159	83	25	50	318	22	19	676
Proportion within study population (%)	23.5	12.3	3.7	7.4	47.0	3.3	2.8	100%
Age*, years Mean (SD)	38.8 (18.2)	50.9 (19.2)	35.9 (12.3)	66.3 (17.5)	30.8 (14.2)	34.1 (11.8)	42.4 (16.4)	38.4 (19.0)
Perpetrator’s family relation to the victim** (%)								
Spouses/ex-spouse	55.3	62.7	84.0	44.0	44.1	91.0	84.2	53.0
Father	3.1	1.2	0	4.0	5.6	0	5.3	4.0
Brother	8.8	1.2	4.0	0	14.4	4.5	0	9.3
Son	13.8	16.9	0	26.0	4.1	0	0	9.2
Other relative/s	18.9	18.1	12.0	26.0	31.9	4.5	10.5	24.6
Proportion of pregnant victims** (%)	21.4	4.8	28.0	2.0	25.5	9.1	0	19.1
Injury Severity** (%)								
Mild Injury (ISS 1–8)	73.6	60.2	76.0	46.0	88.4	57.2	73.7	76.4
Moderate Injury (ISS 9–14)	15.7	22.9	4.0	40.0	4.1	19.0	10.5	12.4
Severe and critical Injury (ISS 16 +)	10.7	16.9	20.0	14.0	7.5	23.8	15.8	11.1

*p value of ANOVA test < .05

**p value of χ^2^ test < .05

*IBJ* Israeli born Jews, *IFSU* Immigrants from Former Soviet Union, *EI* Immigrants from Ethiopia, *OI* Other Jewish Immigrants, *IA* Israeli Arabs, *FW* Foreign workers and tourists

**Fig. 1:**
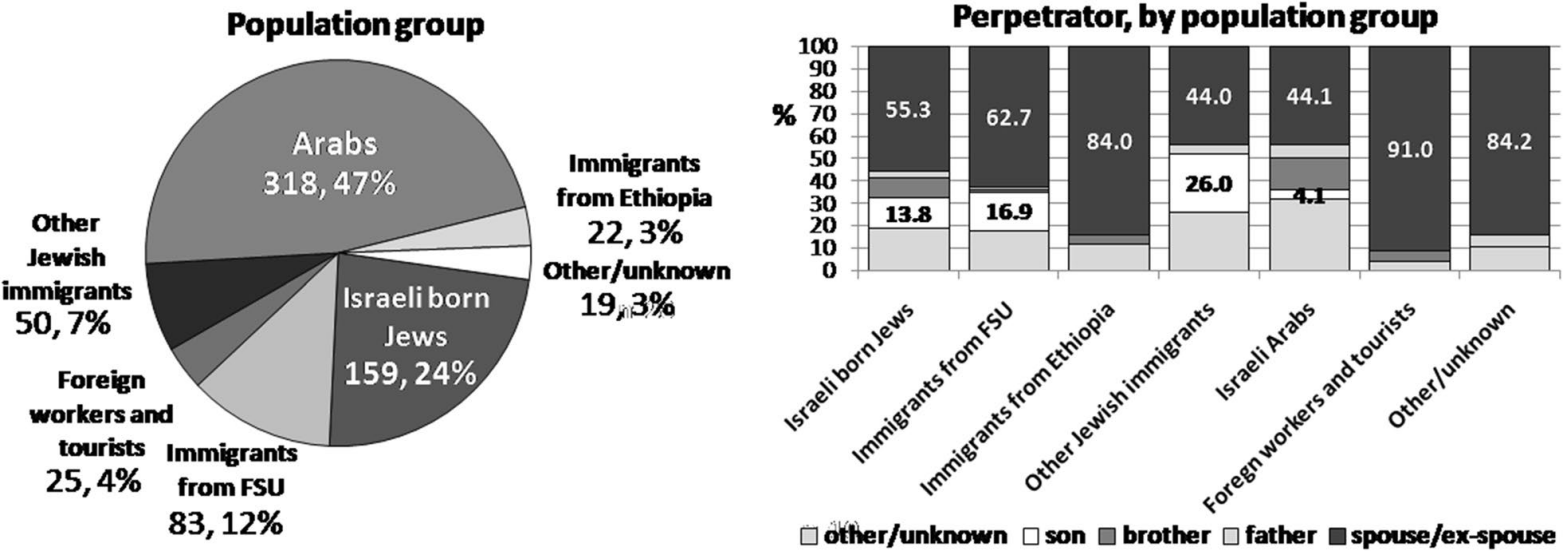
Study population (n = 676)

In most cases, the spouse or ex-spouse caused the injury. The family relation with the perpetrator was distributed differently between the population groups (Table 1 and Fig. 1). The proportion of brothers who attacked sisters was greatest among Arabs (14.4%), while the phenomenon of attacking a mother was very rare in the Arab sub-group. In contrast, among Jewish women, the proportion of those injured by a son was high, especially among the group of OI (26.0%) and among immigrants from FSU (16.9%).

Among the hospitalized victims, 19.1% (n = 129) were pregnant when they were attacked (see Table 1 and Fig. 1). The highest prevalence rate of pregnant casualties was observed among EI (28.0%) and among Israeli Arabs (25.5%). Pregnant casualties had significantly higher frequency of mild injuries (ISS 1–8)—98% in comparison with non-pregnant (71%) (*p* < 0.0001). Among the pregnant casualties, the most frequent perpetrator was the spouse or ex-spouse (60%). The proportion of at least moderate injuries was higher among OI (54%), immigrants from FSU (39.8%), and foreign workers and tourists (40.9%).

While the majority (77.4%, n = 523) of women were beaten, 13.3% (n = 90) were stabbed, 1% (n = 6) were shot, and among 8.3% (n = 57), the mechanisms of injury included pushing from higher grounds, burns, human bites or run over with a vehicle. Most casualties (n = 564, 83.4%) sustained a blunt injury, 14.3% sustained penetrating injuries, and 2.2% suffered from burns.

**Fig. 2:**
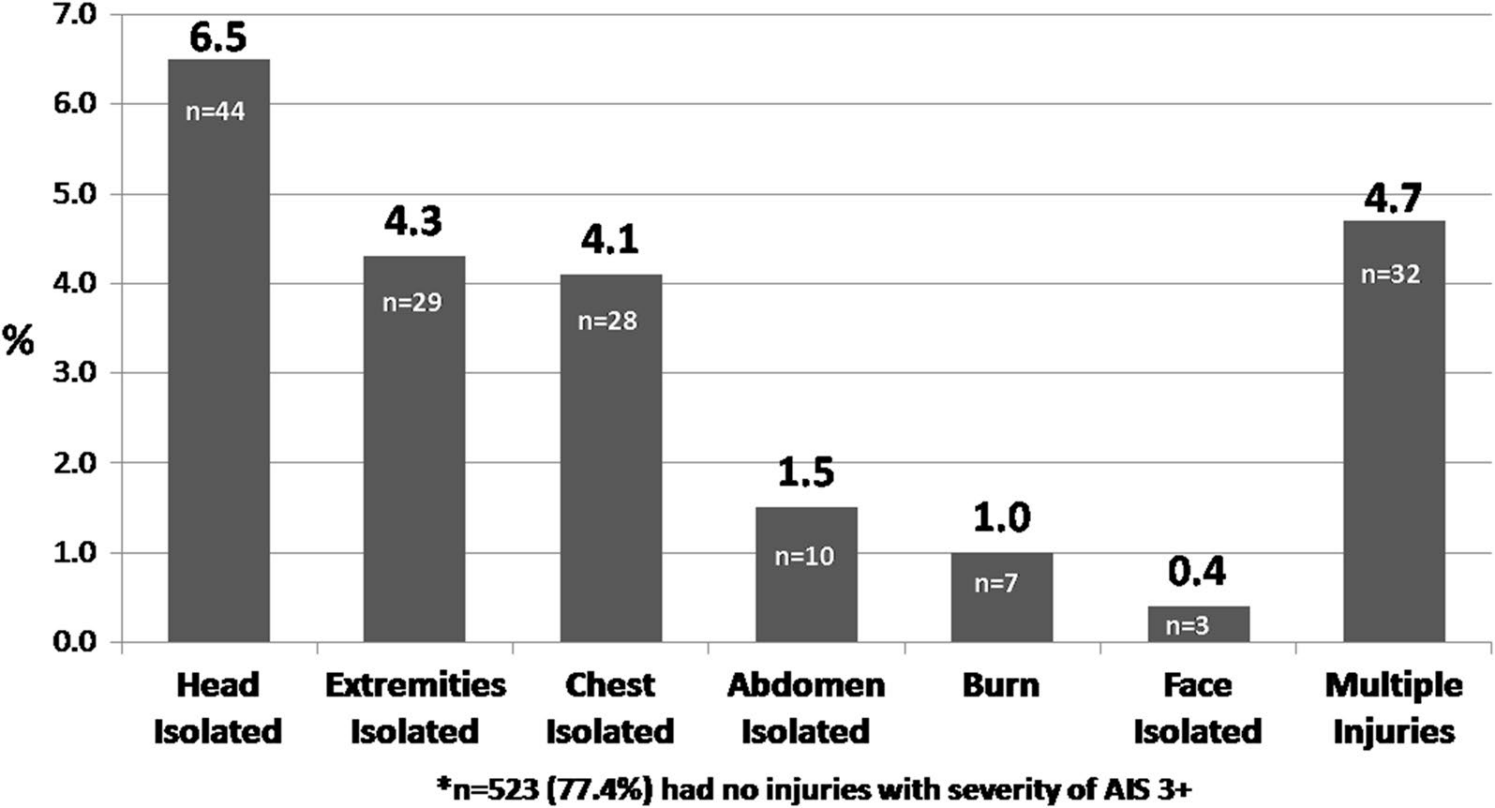
Frequency of severe injury (AIS 3+) by body region*

The majority of injuries (77.4%, n = 523) were not severe (AIS < 3). The frequency of severe injuries by body region is depicted in Fig. 2.

The most frequent isolated severe injury was a head injury, in which 6.5% of the women (n = 44) suffered from a severe (AIS 3+) isolated head injury. Almost 5% of women suffered from multiple severe injuries. While the mean age of abused hospitalized women was 38.4, the mean age for mothers attacked by a son was 59.4 years, the mean age of girls who were assaulted by their fathers was 18.8 years, and the age range of females who were attacked by other family members was 26.5–38.3, (see Table 2). Almost 22% of the women who were attacked by their spouse or ex-spouse were pregnant.

**Table 2:** Hospitalized victims of domestic violence, demographic and injury characteristics by relation with perpetrator, 2011–2020

	Spouses/ex-spouse	Father	Brother	Son	Other relative/s	Total
n	358	27	63	62	166	676
Proportion among study population	53.0%	4.0%	9.3%	9.2%	24.6%	100%
Age*, year Mean (SD)	38.3 (16.9)	18.8 (4.6)	26.5 (13.9)	59.4 (17.1)	38.4 (19.9)	38.4 (19.0)
Proportion of pregnant victims ** (%)	21.5	0	9.5	6.5	25.3	19.1
Injury Severity Score (ISS) (%)^NS^						
Mild (ISS 1–8)	73.1	85.2	87.3	64.6	82.5	76.4
Moderate (ISS 9–14)	13.4	0	6.3	17.7	12.7	12.4
Severe (ISS 16+)	13.4	14.8	6.3	17.7	4.8	11.1
Hospitalization in ICU (%)^NS^	9.2	11.1	4.8	8.1	2.4	7.1
Length of Stay***, median [IQR]	2.0 [1–4]	1.0 [1–5]	2.0 [1–3]	2.5 [1–7.25]	1.0 [1–3]	2.0 [1–4]
Undergoing Surgery** (%)	18.7	14.8	17.5	25.8	10.2	17.0
In-hospital mortality (%)^NS^	2.0	3.7	0	4.8	1.2	1.9

*p value of ANOVA test < .05

**p value of χ^2^ test < .05

***p value of Kruskal Wallis test < .05

^**N**^^**S**^χ^2^ test showed non-significant results

Injury severity and use of hospital resources differed among the victims. Mothers who were injured by their sons suffered from the most severe injuries (35% of women had a moderate/severe/critical injury). In comparison to other victims, mothers experienced the most extended hospital stays (median 2.5 days for mothers compared to 1–2 days for other victims), a greater need for surgery (25.8% for mothers vs. 10.2–18.7% for other victims), and the highest in-hospital mortality rate (4.8% for mothers vs. 1.2–3.7% for other victims).

In a multivariable logistic regression model with at least moderate injury as a dependent variable, trauma type was significantly associated with odds for suffering from at least one moderate injury, after adjustment for age, trauma type, population group, and perpetrator`s relation to the victim (see Table 3).

**Table 3:** Odds for at least moderate injury, by demographic and injury characteristics

Characteristics	n	ISS 9 + (n, %)	Odds for injury with ISS 9 +	
	676	159 (23.5%)	Odds ratio (OR)	95% CI
Age, years	–	–	**1.034**	**1.021–1.048**
Trauma type				
Penetrating	97	51 (52.6%)	**6.911**	**4.138–11.570**
Burn	15	6 (40.0)	**5.177**	**1.611–16.636**
Blunt	564	102 (18.1%)	Ref	–
Population group				
Israeli born Jews	159	42 (26.4%)	**2.140**	**1.237–3.701**
Immigrants from FSU	83	33 (39.8%)	**2.862**	**1.487–5.509**
Immigrants from Ethiopia	25	6 (24.0%)	1.401	0.465–4.225
Other Jewish immigrants	50	27 (54.0)	**4.473**	**2.014–9.935**
Foreign workers and tourists	22	9 (40.9%)	**3.974**	**1.422–11.102**
Other/unknown	19	5 (26.4%)	1.831	0.578–5.802
Israeli Arabs	318	37 (11.6%)	Ref	–
Relation of perpetrator to victim				
Spouse/ex-spouse	358	96 (26.8%)	1.465	0.610–3.521
Father	27	4 (14.8%)	1.503	0.370–6.114
Son	62	22 (35.5%)	0.891	0.307–2.586
Other/unknown	166	29 (17.5%)	0.888	0.343–2.302
Brother	63	8 (12.7%)	ref	-

Bold values indicate statistically significance

Casualties with penetrating trauma had almost sevenfold odds (OR = 6.911, 95% CI 4.138–11.570), and casualties with burns had fivefold higher odds (OR = 5.177, 95% CI 1.611–16.636) for moderate to critical injuries (ISS 9+). The relation of the perpetrator to the victim was not significantly associated with moderate-critical injuries. In comparison to Israeli Arabs (the group with the lowest odds for injury with ISS 9+), Jews had a higher odds for sustaining at least moderate injuries, while the odds of OI (Jewish immigrants not from FSU or Ethiopia) were the highest (OR = 4.473, 95% CI 2.014–9.935).

This model explained 30% in variance in probability to have at least moderate injury.

In 2019, 28 Jewish and 34 Arab women aged 14+ were hospitalized following a domestic violence event. Thus, the annual hospitalization risk was 1.0/100,000 for Jewish women and 5.2/100,000 for Arab women (RR = 5.0, 95% CI 3.0–8.3).

In 2020, 34 Jewish and 38 Arab women were hospitalized following domestic violence; thus, the annual hospitalization risk was 1.3/100,000 and 5.8/100,000, respectively for Jews and Arabs (RR = 4.6, 95% CI 2.9–7.3).

In comparison to 2019, during 2020, the risk of being hospitalized following a domestic violence event increased by 30% for Jewish women and by 12% for Arab women (the association was not significant: RR = 1.30, 95% CI 0.7–2.0 for Jewish women and RR = 1.12, 95% CI 0.7–1.8 for Arab women).

## Discussion

The aim of the current study was to shed light on the scope of domestic violence-related hospitalizations among women in Israel. The outcomes show a higher risk among Arab women in comparison with Jewish women, with a non significant increase from 2019 to 2020 (the outbreak year of the Covid-19 pandemic). The highest probability to sustain moderate and severe injuries (after adjusting for age and other characteristics) was observed among Jewish immigrants from the FSU, other Jewish immigrants (OI), and foreign workers and tourists.

It should be taken into account that the data provided for this study represents an underestimation of the real risk since women often underreport abuse to hospital staff. Abbott [35]found that only 13% of abused women who visited the ED reported to the staff about being the victim of abuse. This low report rate is related to fear that the medical staff would report the problem to the authorities [36]. While women often hesitate to report violence, health care professionals often fail to suspect domestic violence and detect only 5% of battered women. For many abused women, the ED is the first and sometimes the only contact they have with health care clinicians [37]. Even if all women seeking care would be ready to report acts of domestic violence in the ED, these data will most likely represent only the most severe cases. In Western countries, it is estimated that about 25% of women experience intimate partner violence over their lifetimes [38]. In a research by Grynbaum et al. [39], screening of women who visited a primary care setting in Israel showed that lifetime prevalence of family violence is 30.8%, while 10% suffered from violence during the previous year.

A research by Fisher et al. [40] found that among non-pregnant women visiting a women`s health center clinic in northern Israel, 16% suffered minor physical abuse, and 8% suffered severe physical abuse. Likewise, among pregnant women, 20% sustained minor and 8% suffered severe physical abuse. Physical abuse directed to the abdomen (fetus-directed violence) was reported by 5% of pregnant women [40]. In our study, a fifth of hospitalized casualties were pregnant. However, it should be noted that it is recommended to hospitalize pregnant women for at least one day for observation, irrelevant to the type or severity of the injury, thus contributing to a higher hospitalization rate among pregnant women in comparison to non-pregnant women.

Following a high prevalence of domestic violence and its consequences for women, the detection ability of abused women among healthcare professionals should be improved. Of those victims of domestic violence seeking care, over 80% solicit care in a hospital, while others may seek care in other health professional settings [41]. Regardless of detection, there is a need for a comprehensive, multifaceted programs to provide relief to victims if we aspire to effectively combat the problem. Detection should be preceded by national and local efforts to prevent gender-based violence and followed by structured, multidimensional, and tailored interventions focused on helping victims of such violence. The collaboration between police, social and health services, and local authorities is crucial to preventing and treating this phenomenon [42]. Almost 76% of cases filed by the police following a domestic violence event in Israel are closed prior to court hearings, with the most frequent ground for case closing (87%) is insufficient evidence [31]. Thus, not only the victim of abuse often needs to gather and provide the police with sufficient proof of the crime, but also she and her children are often evacuated to a shelter and displaced from their everyday life. In contrast, the perpetrator will usually continue to live in his same apartment and carry on with his everyday routine, often without punishment. Circumstances leading to cases being not fully investigated and punished can often lead the perpetrators to continue his abusing behavior and pose an increased risk to his family members [43]. Indeed, unpunished perpetrators usually continue to abuse their victims [44], and this abuse may contribute not only to a greater risk of physical injury but also a deterioration in health status, chronic pain conditions, substance abuse, reproductive disorders, higher level of depression, anxiety and phobias, eating and sleep disorders, post-traumatic stress disorder, injury-related disability, self-harm, and in extreme cases death and suicide of the victim [44]. Furthermore, children exposed to violence toward their mother often suffer from emotional, mental, and social impairment that can affect developmental growth [45]. A strong association has been found between childhood experiences of abuse and the perpetration or experience of violence against women in adults [46].

Our findings that Arab women, in comparison to Jewish women, are at greater risk for hospitalization due to domestic violence support previous studies with similar outcomes in which Arab women are at high risk for abuse [17, 18, 20, 47]. In Israel, Arab women aged 14 and above comprise 19% of the female population [34]; however, their proportion among hospitalized victims of domestic violence in this study was 47%. Other studies also reported a higher risk of abuse among Arab women in comparison to Jewish women [17, 18, 20, 47]. The phenomenon of a higher frequency of violence against Israeli Arab women is related to several factors. The Arab minority in Israel is predominantly a patriarchal society abiding by attitudes that may promote violence against women [17]. The Muslim community regulates its unique court system and handles marriage and divorce under Islamic law. Eight regional Islamic law courts and one national appeals court operate in Israel under the supervision of Israel's Ministry of Justice. Religiosity was found as one of the important factors significantly associated with higher IPV among Arab women in addition to younger age and low income [18].

Social Learning Theory [48] adds another explanation to higher rates of violence against women in Arab society. In a study by Haj-Yahia and Dawud-Noursi [49], it was reported that 18% of Arab adolescents were witnesses of father physical violence toward the mother during the past 12 months. When children become witnesses of domestic violence, they are susceptible to adopting violent tactics of resolving domestic conflicts; thus, domestic violence is transmitted from generation to generation [49]. Arab women have been reported to experience and witness more violence during their childhood compared to Jewish women [17].

Less family support provided to Arab women in the case of domestic violence [17], may explain their unwillingness to report these cases. In addition, given their minority status, there is a tendency in Arab society to avoid contact with the police [17]. Arabs in Israel view the police more negatively than Jews, and these findings are consistent with a large body of research on racial and ethnic group relations with the police as well as the specific literature concerning deeply divided societies [50].

Finally, significant socio-economic differences exist between the Jewish majority of the population in Israel and Arab-Israelis, who often live in separate towns, villages or neighborhoods [51]. The low socio-economic position is a known risk factor for domestic violence [52] independently associated with a higher rate of domestic violence [8]. Improvements in the socio-economic position of Arab women are expected to be more prevalent, as Arab women today qualify for a matriculation certificate at rates approaching those of Jewish women. In addition, there is a notable increase in higher education rates among Arab women. However, the lack of daycare centers, public transport, strong traditional norms, and limitations in Hebrew language fluency has led many of these women to choose the field of education, which is seen as more “feminine” and allows them to work within their community at hours convenient for mothers [53]. As the socioeconomic status of Arab women improves, there is hope for improvement in the status of women in the Arab community, which could hopefully lead to a decrease in family violence.

Another group which has a higher representation in the study population than in the general population of Israeli women is the population of Jewish immigrants [54]. It is expected that particularly new immigrant families would be more susceptible to increased aggression due to the mens’ attempts to regain their lost social status [55]. Attacked women in this sub-population were older (but this difference can not explain the higher injury severity in this group as adjustment for age was conducted), we believe that other differences were not taken into account, such as existence of comorbidities, as the data was not available in this database. One of the factors related to the abuse of older women by a family member could be due to crowded living conditions [56, 57]. The situation in which adult children live with their parents is more common among the immigrants from FSU [58], explaining the greater incidence of violent encounters exhibited by a son toward his mother. In addition, it was previously found that one-third of the femicide cases among the immigrants from FSU were committed under the influence of alcohol [20]; that is why it is logical to suppose that alcohol may be involved in initiating violence.

Among the Ethiopian casualties, the proportion of spouse or ex-spouse as an abuser is very high. The Ethiopian community is predominantly a patriarchal religious community, which has experienced many hardships related to absorption and adjustment into Israeli society. Ethiopian men in Ethiopia were the head of the family and well respected in the traditional Ethiopian patriarchal system. However, after arriving in Israel, many of them became unemployed or worked in low-income jobs and often lost their sense of power, honor, or respect [59]. Kacen [60] wrote in her ethnographic study about spousal abuse among Ethiopian immigrants in Israel: "During the cultural transition, the immigrants' code of honor, traditional conflict-solving institutions, and family role distribution disintegrate. This situation, exacerbated by economic distress, proved conducive to women's abuse". According to previous studies, the community support for Ethiopian women in the case of family violence is very low [61].

Additional findings from the study, support the data showing an increase in domestic violence during the lockdowns associated with the Covid-19 pandemic, although this increase was not statistically significant. In Israel, from 2019 to 2020, there was an increase of about 28% in the number of reported domestic violence cases involving Jewish women, and an increase of 10% in the number of violent offenses against non-Jewish women in Israel [62]. In several other countries, the increases in emergency calls reporting domestic violence have been observed [63]. Violence against women tends to increase in any emergency, including epidemics [5, 64]. Stress, disruption of social and protective networks, increased economic hardship, and decreased access to services can exacerbate women's risk of suffering domestic violence [63]. Economic instability following the lockdowns, partially expressed by the doubling of unemployment rates that reached 27.8% [65], as well as lack of safe and stable childcare due to lockdowns, worsened already tenuous situations during Covid-19 related lockdowns [66].

## Study limitations

Data regarding all sub-groups of the Jewish population is not available in the INTR; thus, it was impossible to study the scope of violence-related hospitalization among the various religious sectors. In addition, SES and education level are not available in INTR data; thus, it was not possible to adjust ethnic differences to SES and/or education as possible confounding associations exist between the ethnic group and family violence characteristics.

Finally, as exact number of tourists and foreign nationals in Israel and their lengh of stay in the country are unknown, annual risk was not calculated among this group.

## Policy implications and recommendations

The Ecological Model [4] suggests identifying and minimizing the risk factors at every level (individual, family, community, and society) through prevention and treatment interventions for putting a stop to violence against women [67]. Interventions should identify and reinforce protective factors, such as decreasing the likelihood of women and girls to encounter violence, at each level within the ecological model. In Israel, domestic violence prevention should focus on populations at risk, including Arab women, new immigrants, and women living in low socioeconomic status areas. These women are especially vulnerable and often lack sufficient family support and resources to escape the trap of domestic violence. Among Arabs and Ethiopian women, it is essential to develop and strengthen community-based social support services available for female victims of violence. On the other hand, as the phenomenon of domestic violence is cross-sectoral, improvements in prevention and treatment should be made throughout the entire Israeli society.

Programs should be mindful of the different levels in the ecological model to achieve the desired results. Each level is interconnected, and choosing interventions at one or more levels will influence the risk and protective factors within other levels.

1. On **the individual level**, children living in domestic violence environments should be identified by educator staff. After identifying these children, who are potentially at risk for becoming abusers or victims in future relationships as grown-ups, they should receive professional counseling [68] to prevent continuation of the chain of violence. The Ministry of Education should be responsible for training educators to address this issue, from identifying children at risk to providing appropriate interventions in their families.

2. On **the family level**, women in low socio-economic communities and minority women should be provided with easy access to higher education and employment opportunities, which aims to empower and provide them independence. For example, a pilot program for Bedouin in the South of Israel enables this minority population to apply to five academic institutions of higher education without a psychometric examination and are provided with additional educational assistance. Similar programs should be implemented for women of other minorities and women of low socio-economic backgrounds. The Council for Higher Education should be in charge of such intervention programs, which encourages higher education.

The outcomes of this study showed that immigrant women are at higher risk of becoming victims of domestic violence. Thus, we recommend that the Ministry of Aliyah (Immigration) and Integration be part of an interdisciplinary team to identify immigrants at risk and provide language and culturally adapted prevention programs.

3. The **community level** should include cooperation with religious leaders. For example, in the Muslim community, the religious leaders (Imams) have significant influence and have the capacity to facilitate positive change, and contribute to health promotion [69]. Thus, they can educate members of the community regarding the importance of engaging in non-violent behaviors, customs, and practices in the home, in schools, and between couples [70].

4. T**he societal level** should include the implementation of appropriate laws, legislations, and policies. The police should improve their response towards victims of domestic violence, enforce all legislation protecting abused women and restricting abusers.

Ministry of Health should invest in appropriate training to increase the knowledge and skill sets of medical staff in the effort of identifying the victims of domestic violence and access services dedicated to providing the necessary support and treatment for them.

Effective intervention programs should be multidisciplinary and include a variety of professionals, policy makers and representatives from various ministries (police, the court system, social and health services, and local authorities [71–78]. Findings regarding such interdisciplinary cooperation programs which incorporate response of different authorities to the domestic violence phenomenon showed promising improvements [75–79]. In such multidisciplinary cooperation, professionals from different agencies should, on the one hand, be aware of the core tasks, duties, responsibilities of every agency, but on another hand be ready to cross professional boundaries, as it is essential for genuine and flexible collaboration [80]. Public Health professionals should serve as coordinators of such strategies and mediators between the different agencies. Implementation of such future projects should be followed by assessment of its effectiveness. Although such follow-up is expected to be a challenging task, it is essential for future improved outcomes.

## Data Availability

Data sharing not applicable to this article as the ITR does not allow any files to be released.

## References

[1] Dobash RE, Dobash RP, Dobash RE, Dobash RP (2012). Violent Men and Violent Contexts. Rethinking violence against women.

[2] Fox VC (2002). Historical perspectives on violence against women. J Int Womens Stud.

[3] Belsky J (1980). Child maltreatment: an ecological integration. Am Psychol.

[4] Heise LL (1998). Violence against women: an integrated, ecological framework. Violence Against Woman.

[5] Rubenstein BL, Lu LZN, MacFarlane M, Stark L (2020). Predictors of interpersonal violence in the household in humanitarian settings: a systematic review. Trauma Violence Abuse.

[6] Krug EG, Mercy JA, Dahlberg LL, Zwi AB (2002). The world report on violence and health. Lancet.

[7] Rocca CH, Rathod S, Falle T, Pande RP, Krishnan S (2009). Challenging assumptions about women’s empowerment: social and economic resources and domestic violence among young married women in urban South India. Int J Epidemiol.

[8] Oguntayo R, Oyeleke JT, Popoola OA, Opayemi AS, Faworaja OR (2018). Influence of socio-economic factors on domestic violence among couples in Ibadan metropolis. ESUT J Psychol Sci..

[9] Wilcox, Annika M. A study of domestic violence and patriarchal ideologies in popular men’s magazines. 2015.

[10] Injury Center|CDC. Preventing intimate partner violence |violence prevention|. https://www.cdc.gov/violenceprevention/intimatepartnerviolence/fastfact.html. Published 2020. Accessed 13 Apr 2021.

[11] Hawcroft C, Hughes R, Shaheen A (2019). Prevalence and health outcomes of domestic violence amongst clinical populations in Arab countries: a systematic review and meta-analysis. BMC Public Health.

[12] Gross N. Israel’s patriarchal culture. The New York Times. https://www.nytimes.com/2010/10/19/opinion/19iht-edlet.html. Published 2010.

[13] Harris E. In Israel, Jewish Divorce is granted only by husband’s permission. WBAA. https://www.wbaa.org/post/israel-jewish-divorce-only-granted-husbands-permission#stream/0. Published 2015.

[14] Heller, A., Hazboun A. Killings spark reckoning over status of Arab women in Israel. The Times of Israel. https://www.timesofisrael.com/killings-spark-reckoning-over-status-of-arab-women-in-israel/. Published 2016.

[15] CBS. Israel’s Independence Day 2019. 2019:6–8. https://www.cbs.gov.il/he/mediarelease/DocLib/2019/134/11_19_134b.pdf. Accessed 14 May 2021.

[16] Zaatut A, Haj-Yahia MM (2016). Beliefs about wife beating among Palestinian women from Israel: the effect of their endorsement of patriarchal ideology. Fem Psychol.

[17] Ben-Porat A, Levy D, Kattoura O, Dekel R, Itzhaky H (2021). Domestic Violence in Arab society: a comparison of Arab and Jewish women in shelters in Israel. J Interpers Violence.

[18] Daoud N, Sergienko R, Shoham-Vardi I (2020). Intimate partner violence prevalence, recurrence, types, and risk factors among Arab, and Jewish immigrant and nonimmigrant women of childbearing age in Israel. J Interpers Violence.

[19] Shoval Y (2000). Social structure and health in Israel.

[20] Sela-Shayovitz R (2010). The role of ethnicity and context: intimate femicide rates among social groups in Israeli society. Violence Against Women.

[21] The Population of Israel. The Israeli Bureau of Statistics; 2018. http://www.cbs.gov.il/reader/?MIval=cw_usr_view_SHTML&ID=629. Accessed 14 May 2021.

[22] Cohen JB (2016). Ethiopian-Israeli community. BMJ Case Rep.

[23] Tiruneh A, Siman-Tov M, Radomislensky I (2017). Characteristics and circumstances of injuries vary with ethnicity of different population groups living in the same country. Ethn Health.

[24] Tiruneh A, Radomislensky I, Peleg K, Siman-tov M, ITG (2019). Minorities and foreign born are disproportionately affected by injuries due to violence: an analysis based on a National Trauma Registry 2008–2017. Isr J Health Policy Res.

[25] Edelstein A (2013). Culture transition, acculturation and intimate partner homicide. SpringerPlus.

[26] Joffre T. Thousands protest violence against women across Israel after murders. The Jerusalem Post. https://www.jpost.com/israel-news/thousands-protest-violence-against-women-across-israel-after-murders-646467. Published 2020.

[27] Murder of Diana Raz. Arutz Sheva. https://www.israelnationalnews.com/News/News.aspx/297495. Published 2021.

[28] Woman brutally beaten by husband. Aritz Sheva. https://www.israelnationalnews.com/News/News.aspx/290137. Published 2021.

[29] Tatiana was murdered in her apartment in Sderot. ynet. https://www.ynet.co.il/news/article/syupvoigf. Published 2021.

[30] Lanzkron N. The women murdered in Israel in 2018. The Times of Israel. https://www.timesofisrael.com/the-24-women-murdered-in-israel-since-the-start-of-the-year/. Published 2018.

[31] Yachimovich-Cohen N. Domestic violence in Israel: the police data 2016–2017. https://m.knesset.gov.il/EN/activity/mmm/DomesticViolenceWithanemphasisonViolenceAgainstWomen.pdf. Accessed 14 May 2021.

[32] Deng Q, Tang B, Xue C (2016). Comparison of the ability to predict mortality between the injury severity score and the new injury severity score: a meta-analysis. Int J Environ Res Public Health.

[33] Copes WS, Champion HR, Sacco W (1988). The injury severity score revisited. J Trauma.

[34] Central Bureau of Statistics*. *Israeli population, 2019. 2020. https://www.cbs.gov.il/he/publications/Pages/2020/אוכלוסייה-שנתון-סטטיסטי-לישראל-2020-מספר-71.aspx.

[35] Abbott J (1995). Domestic violence against women. Incidence and prevalence in an emergency department population. JAMA J Am Med Assoc.

[36] Hayden SR, Barton ED, Hayden M (1997). Domestic violence in the emergency department: how do women prefer to disclose and discuss the issues?. J Emerg Med.

[37] Mazza D, Lawrence JM, Roberts GL, Knowlden SM (2000). What can we do about domestic violence?. Med J Aust.

[38] Gracia E (2004). Unreported cases of domestic violence against women: towards an epidemiology of social silence, tolerance, and inhibition. J Epidemiol Community Health.

[39] Grynbaum M, Biderman A, Levy A, Petasne-Weinstock S (2001). Domestic violence: prevalence among women in a primary care center—a pilot study. Isr Med Assoc J.

[40] Fisher M, Yassour-Borochowitz D, Neter E (2003). Domestic abuse in pregnancy: results from a phone survey in Northern Israel. Isr Med Assoc J.

[41] Huecker MR, King KC, Jordan GA, Smock W (2021). Domestic violence.

[42] National Institute of Clinical Excellence. Domestic violence and abuse: how health services, social care and the organisations they work with can respond effectively. NICE Public Health Guid. 2014;50(February):0–72.

[43] Moe AM (2007). Silenced voices and structured survival: battered women’s help seeking. Violence Against Women.

[44] Rakovec-Felser Z (2014). Domestic violence and abuse in intimate relationship from public health perspective. Heal Psychol Res.

[45] Kulka T, Padilha MGS, Antunes MC (2020). Effects of domestic violence against women on their children. Trends Psychol.

[46] Fry DA, Elliott SP (2017). Understanding the linkages between violence against women and violence against children. Lancet Glob Heal.

[47] Shwartz N, O’Rourke N, Daoud N (2020). Pathways linking intimate partner violence and postpartum depression among Jewish and Arab women in Israel. J Interpers Violence.

[48] Copp JE, Giordano PC, Longmore MA, Manning WD (2019). The development of attitudes toward intimate partner violence: an examination of key correlates among a sample of young adults. J Interpers Violence.

[49] Haj-Yahia MM, Dawud-Noursi S (1998). Predicting the use of different conflict tactics among Arab siblings in Israel: a study based on social learning theory. J Fam Violence.

[50] Hasisi B, Weitzer R (2007). Police relations with Arabs and Jews in Israel. Br J Criminol.

[51] OECD economic surveys: Israel 2020. Paris: OECD; 2020. 10.1787/d6a7d907-en.

[52] Capaldi DM, Knoble NB, Shortt JW, Kim HK (2012). A systematic review of risk factors for intimate partner violence. Partn Abus.

[53] Fuchs H. Education and employment among young Arab Israelis. *Taub Cent*. 2017;(December):1–50. http://taubcenter.org.il/wp-content/files_mf/arabisraelisinhighereducation.pdf. Accessed 14 May 2021.

[54] Razin A (2018). Israel immigration story: winners and losers. Isr Econ Rev.

[55] Eisikovits Z, Winstok Z, Fishman G (2004). The first Israeli national survey on domestic violence. Violence Against Women.

[56] World Health Organization. Elder abuse. No. WPR/2016/DNH/010. Manila: WHO Regional Office for the Western Pacific, 2016.

[57] Luoma ML, Koivusilta M, Lang G, Enzenhofer E, Donder L, Verté D, Reingarde J, et al. Prevalence study of abuse and violence against older women: results of a multi-cultural survey conducted in Austria, Belgium, Finland, Lithuania, and Portugal. National Institute for Health and Welfare, 2011.

[58] Korenman-Iluz M. Social immigration of FSU citizens from Israel to the United States. 2019.

[59] Jaffe A, Guttman N, Schuster M. The evolution of the Tene Briut model: Developing an intervention program for the Ethiopian immigrant population in Israel and its challenges and implications. En Epstein L (ed.) (2007):121–142.

[60] Kacen L (2006). Spousal abuse among immigrants from Ethiopia in Israel. J Marriage Fam.

[61] Lilach Y. Ethiopian battered women in shelters: the contribution of characteristics of violence, one’s life events and personal and social resources to the variation of mental distress | School of Social Work. 2016. https://social-work.biu.ac.il/en/node/3259. Accessed 11 Apr 2021.

[62] Domestic violence in Israel up 300% during coronavirus pandemic. https://www.ynetnews.com/article/S1mLRYjcw. Published 2020. Accessed 14 May 2021.

[63] Ertan D, El-Hage W, Thierrée S, Javelot H, Hingray C (2020). COVID-19: urgency for distancing from domestic violence. Eur J Psychotraumatol.

[64] Molyneaux R, Gibbs L, Bryant RA (2020). Interpersonal violence and mental health outcomes following disaster. BJPsych Open.

[65] Israeli Employment Service. Monthly update on the workforce in Israel. 2020. https://www.taasuka.gov.il/he. Accessed 14 May 2021.

[66] Evans ML, Lindauer M, Farrell ME (2020). A pandemic within a pandemic—intimate partner violence during Covid-19. N Engl J Med.

[67] Operating within the ecological model. UN Women. https://www.endvawnow.org/en/articles/310-operating-within-the-ecological-model-.html. Published 2010. Accessed 24 July 2021.

[68] Davis J, Briggs E. Witnessing violence fact sheet. Medical University of South Carolina. https://mainweb-v.musc.edu/vawprevention/research/witnessing.shtml. Published 2000. Accessed 24 July 2021.

[69] Mustafa Y, Baker D, Puligari P, Melody T, Yeung J, Gao-Smith F (2017). The role of imams and mosques in health promotion in Western societies: a systematic review protocol. Syst Rev.

[70] Prezenszky BC, Galli EF, Bachega D, de Mello RR (2018). School actions to prevent gender-based violence: a (quasi-)systematic review of the Brazilian and the international scientific literature. Front Educ.

[71] Mazerolle L, Eggins E, Sydes M, Hine L, McEwan J, Norrie G, Somerville A. Criminal justice responses to domestic and family violence. 2018.

[72] Logar R, Vargová BM. Effective multi-agency co-operation for preventing and combating domestic violence training of trainers manual; 2015. http://fileserver.wave-network.org/home/Training_Trainers_Manual_2016.pdf. Accessed 14 May 2021.

[73] Logar R. The Austrian model of intervention in domestic violence cases; 2005. http://www.un.org/womenwatch/daw/egm/vaw-gp-2005/docs/experts/logar.dv.pdf. Accessed 14 May 2021.

[74] Phillips J, Dunkley A. Domestic violence: issues and policy challenges—parliament of Australia; 2015. https://www.aph.gov.au/About_Parliament/Parliamentary_Departments/Parliamentary_Library/pubs/rp/rp1516/DVIssues. Accessed 17 Sept 2021.

[75] Vogt C (2020). Interagency cooperation. Eur Law Enforc Res Bull.

[76] Pfleiderer B. Development of online training platform on domestic violence for police, health care and social sector. October 2020. https://apha.confex.com/apha/2020/meetingapp.cgi/Paper/486783. Accessed 26 Sept 2021.

[77] Machado P, Pais LG, Morgado S, Felgueiras S (2021). An inter-organisational response to domestic violence. Eur Law Enforc Res Bull.

[78] Wojcik MLT, Rubenstein BY, Petkus AA (2021). Coming together in the fight against intimate partner violence: lessons learned from a researcher-practitioner collaboration evaluating Cincinnati’s domestic violence enhanced response team (DVERT). J Contemp Crim Justice.

[79] Cutrone J. Multisite evaluation of the multidisciplinary team (MDT) approach to violence against women in illinois. 2013.

[80] Notko M, Husso M, Piippo S, Fagerlund M, Houtsonen J (2021). Intervening in domestic violence: interprofessional collaboration among social and health care professionals and the police. J Interprof Care.

